# Distinct retroelement classes define evolutionary breakpoints demarcating sites of evolutionary novelty

**DOI:** 10.1186/1471-2164-10-334

**Published:** 2009-07-24

**Authors:** Mark S Longo, Dawn M Carone, NISC Comparative Sequencing Program, Eric D Green, Michael J O'Neill, Rachel J O'Neill

**Affiliations:** 1 Center for Applied Genetics and Technology, Department of Molecular and Cell Biology, University of Connecticut, Storrs, CT 06269, USA; 2 Genome Technology Branch and NIH Intramural Sequencing Center (NISC), National Human Genome Research Institute, National Institutes of Health, Bethesda, MD 20892, USA; 3 Department of Molecular and Cell Biology, University of Connecticut, Storrs, CT 06269, USA

## Abstract

**Background:**

Large-scale genome rearrangements brought about by chromosome breaks underlie numerous inherited diseases, initiate or promote many cancers and are also associated with karyotype diversification during species evolution. Recent research has shown that these breakpoints are nonrandomly distributed throughout the mammalian genome and many, termed "evolutionary breakpoints" (EB), are specific genomic locations that are "reused" during karyotypic evolution. When the phylogenetic trajectory of orthologous chromosome segments is considered, many of these EB are coincident with ancient centromere activity as well as new centromere formation. While EB have been characterized as repeat-rich regions, it has not been determined whether specific sequences have been retained during evolution that would indicate previous centromere activity or a propensity for new centromere formation. Likewise, the conservation of specific sequence motifs or classes at EBs among divergent mammalian taxa has not been determined.

**Results:**

To define conserved sequence features of EBs associated with centromere evolution, we performed comparative sequence analysis of more than 4.8 Mb within the tammar wallaby, *Macropus eugenii*, derived from centromeric regions (CEN), euchromatic regions (EU), and an evolutionary breakpoint (EB) that has undergone convergent breakpoint reuse and past centromere activity in marsupials. We found a dramatic enrichment for long interspersed nucleotide elements (LINE1s) and endogenous retroviruses (ERVs) and a depletion of short interspersed nucleotide elements (SINEs) shared between CEN and EBs. We analyzed the orthologous human EB (14q32.33), known to be associated with translocations in many cancers including multiple myelomas and plasma cell leukemias, and found a conserved distribution of similar repetitive elements.

**Conclusion:**

Our data indicate that EBs tracked within the class Mammalia harbor sequence features retained since the divergence of marsupials and eutherians that may have predisposed these genomic regions to large-scale chromosomal instability.

## Background

Large-scale genome rearrangements, such as translocations, inversions and deletions of chromosomal regions several megabases in length, are characteristic of the genomic instability observed in many different human disease states. For example, jumping translocations often result in tumor-specific chromosome imbalances that are associated with oncogenesis in leukemia [1] and solid tumors [2]. In addition to an association with instability manifest in disease, large-scale rearrangements account for much of the karyotypic diversity observed among species (e.g. [3]). While genome instability and chromosome heterozygosity are often the immediate results of such genomic change [4-6], propagation in the germ line and subsequent fixation leading to species-specific karyotypes are also potential outcomes [7,8]. Each of these specific rearrangements, interchromosomal translocations, deletions or intrachromosomal inversions, requires double stranded breaks. However, the genetic sequences associated with, and mechanisms responsible for, these breaks and rearrangements are not well understood. Tracking these chromosomal rearrangements in both species evolution and disease progression has led to a better understanding of the trajectory and character of chromosome segments during periods of instability.

Nadeau and Taylor [9] proposed that chromosomal breaks associated with rearrangements occurred at random points in the genome. This view has changed as comparative analyses using phylogenetic inference have been performed on the whole-genome sequence data available for several mammalian taxa [10-14]. These genome-wide analyses show that there are many regions, or fragile sites, that are prone to breakage distributed nonrandomly in the mammalian genome [12,15]. Many of these fragile sites are conserved between human and mouse [13] and among such diverse species as rat, cattle, dog, pig, cat, and horse [12] whose evolutionary history spans 95 million years [16]. These data indicate that breakpoint reuse occurs at specific sites in the genome (i.e. EBs). Such genome-scale comparisons show that the fragile regions in one species are often centromeres and/or telomeres at the orthologous region of another species [12].

Recent studies of the evolutionary trajectory of orthologous chromosome segments in Metatherian lineages (Marsupialia) show that EBs often coincide with latent centromeres, locations in the genome that are predisposed to centromere activity [3,4,17]. While both the metatherian [17] and eutherian [12] data suggest an association between EBs and centromeres across diverse vertebrate lineages, it is unknown whether specific sequence motifs are common at both EBs and centromere domains that may indicate shared function. Moreover, common and/or conserved sequence motifs between orthologous EBs shared between eutherian and metatherian lineages have not been previously examined. This study uses sequence data from the tammar wallaby, *Macropus eugenii*, in the context of the kangaroo karyotypic divergence, and from human in the context of primate karyotypic evolution, to explore the possible relationship between these two distinct genomic regions that share a common predisposition to both instability and centromere formation/activity. We hypothesize that active centromeres and EBs identified as latent centromeres are characterized by distinct repeat patterns that are retained during genome restructuring events and that these patterns are a conserved feature of mammalian genomes. Using fluorescence *in situ *hybridization, clone contig assembly, sequence annotation and repeat analyses, we have examined sequence from a conserved EB that has been reused multiple times in the derivation of divergent species karyotypes within the marsupial lineage (EB: Meu1q). These data were compared to genome sequence from centromeric regions (CEN) and euchromatic regions (EU) for conserved features that might indicate a structural and/or functional link among these chromosome domains. In addition, the region of the human genome orthologous to the Meu1q EB was identified as an EB and further analyzed to determine whether specific sequences and/or sequence classes are conserved between metatherian and eutherian lineages during genome reorganization.

Here we report that a high concentration of ERV and L1 elements is shared at centromeres and an EB in the tammar wallaby, as well as at the orthologous EB in human. These results show that the presence of these specific classes of repeat elements are 1) shared at EB that are derived from centromeres, and 2) are conserved at these EB over 180 million years of evolution, despite replenishment with lineage-specific elements. Therefore, the concentration of these elements at EB and centromeres may be a contributing factor to the karyotypic instability these genomic locations have retained.

## Results

### Karyotypically defined regions of the tammar wallaby genome

Previous studies have shown that junctions (heretofore referred to as breaks) between conserved chromosome segments in the tammar wallaby (*Macropus eugenii*) karyotype, as defined by reciprocal chromosome painting [17], carry the kangaroo endogenous retrovirus, KERV [18]. In an effort to expand on the previous breakpoint map, a tammar wallaby bacterial artificial chromosome (BAC) library was screened with the *gag *open reading frame of KERV [18]. From this screen, 49 KERV-containing clones were selected and mapped to metaphase chromosomes using fluorescence *in situ *hybridization (FISH). Of the clones examined, 100% mapped to regions recognized as EBs between conserved chromosome segments or active centromere regions (either pericentric or centric) within the *Macropididae *lineage [17,19].

**Figure 1:**
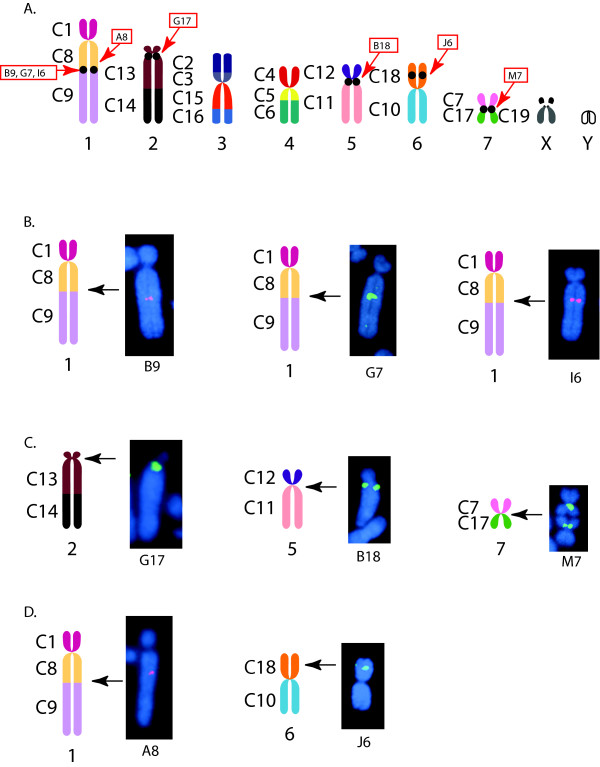
**Fluorescence *in situ *hybridization (FISH) of eight BACs identified in KERV-1 screen**. (A) Tammar karyotype depicting marsupial syntenic segments and cytological localization of BAC clones (as per [22]). (B-D) BACs FISH mapped to tammar metaphase chromosomes localizing to (B) 1q evolutionary breakpoint (EB), (C) centromeres (CEN), and (D) euchromatin (EU).

**Figure 2:**
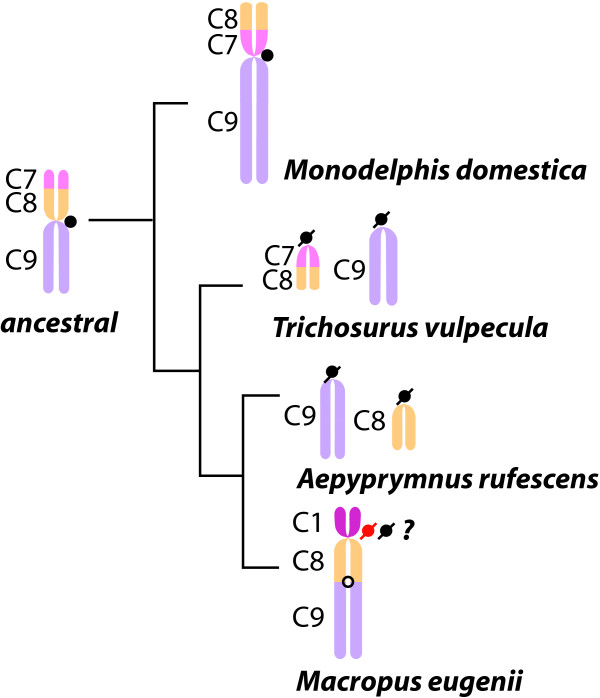
**Phylogenetic trajectory of the chromosome segments participant in the derivation of Meu1q (segments C8 and C9)**. Ancestral orientation is derived from [7] and key species representing 65 million years of marsupial evolution are derived from [17,19,21]. Key is shown to left.

From these KERV-containing clones, three different subsets (see Additional File 1) were selected for finished sequencing and analysis as representatives of specific chromosome regions in the tammar wallaby genome. These include those from a conserved EB, active centromeres (CEN) and euchromatic regions (EU) not involved in karyotypic rearrangements in marsupials (Figure 1A). The CEN locations used in this study are pericentric; however, the pericentric regions within this species are small, with the entire centromere regions spanning only ~420 kb [20]. Thus, we refer to these clones as centromeric, fully aware that they likely encompass these small pericentric regions. Three clones (B9, G7, and I6) localize to tammar chromosome 1q (Figure 1B), a region identified as a major EB within the marsupial lineage and an active centromere in *Monodelphis domestica *(South American opossum), *Trichosurus vulpecula *(brush tailed opossum), and *Aepyprymnus rufescens *(rufous bettong) (Figure 2, [7,17,19,21]). None of these three EB BACs form a contiguous sequence (see Methods). Three clones (B18, G17, and M7) localize to CEN regions of chromosomes 5, 2 and 7, respectively (Figure 1C). Two clones analyzed (A8 and J6) localize to interstitial EU regions that are not defined EBs nor latent centromeres [22] within the marsupial karyotype. A8 localizes to a region adjacent to the EB on tammar chromosome 1q while J6 localizes to the middle of 6p (Figure 1D). An additional ten BACs previously mapped to the genomic region encompassing the cystic fibrosis transmembrane regulator gene (*CFTR*) in tammar wallaby [23] were added to the pool of EU BAC sequences used in these analyses. This region was chosen as it is the only other region of the tammar wallaby that has been fully annotated to date.

**Figure 3:**
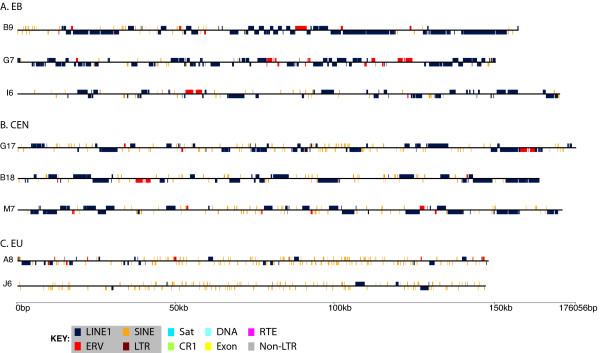
**Annotation of tammar BACs**. BACs for the (A) evolutionary breakpoint (EB: I6, G7, B9) on tammar 1q and (B) pericentric (CEN: B18, G17, M7) regions were annotated to obtain a visual representation of the genomic landscape of each region. Annotations include all predicted interspersed repeats and coding regions. (C) Included, in contrast, are two representations of BACs from euchromatic regions of the tammar genome (A8 and J6). An enrichment of LINE1s (dark blue) and ERVs (red) is seen in both the pericentric and EB with relatively few SINE (orange) elements present. Key to annotated elements is shown at the bottom.

EU, CEN and EB sequences were analyzed for interspersed repeat content and predicted coding regions and the BACs isolated herein were fully annotated for LINEs, SINES, satellites, DNA transposons, RTE elements, endogenous retroviruses (ERVs), LTR retrotransposons, CR1s, non-LTRs, simple repeats and predicted exons. Full annotations for BACs not previously described [23] are shown in Figure 3. Comparison of the relative nucleotide content of the three genomic regions indicates a homogenous distribution with no single nucleotide being more than a fraction of a percent different across these regions. The relative GC content is also uniform across all three regions (EU = 37.73%, CEN = 37.25%, EB = 37.77%). All the identified BACs were analyzed for possible contigs among them using both Multipipmaker and Codon Code Aligner software. No contigs were identified. In addition, it is apparent from the annotation of these BACs (Figure 3) that they do not form a contiguous sequence.

### Enrichment of LINEs and ERVs at tammar wallaby EB and CEN regions

The most striking differences between tammar EU compared with EB and CEN regions are the number and types of repeat elements found as predicted by Repbase's Censor (see Methods)[24]. The total repeat content varies significantly but expectedly, with EU having the fewest repeats (41.3%). Interestingly, the EB carries an even greater number of repeats than the CEN regions, 64.21% and 54.02% respectively (p = 5e^-4 ^and 0.008). A total of 175 different types of repeats were identified. While the abundance of most of these classes (115) did not vary significantly, there are many (60) that did (see Additional File 2).

**Figure 4:**
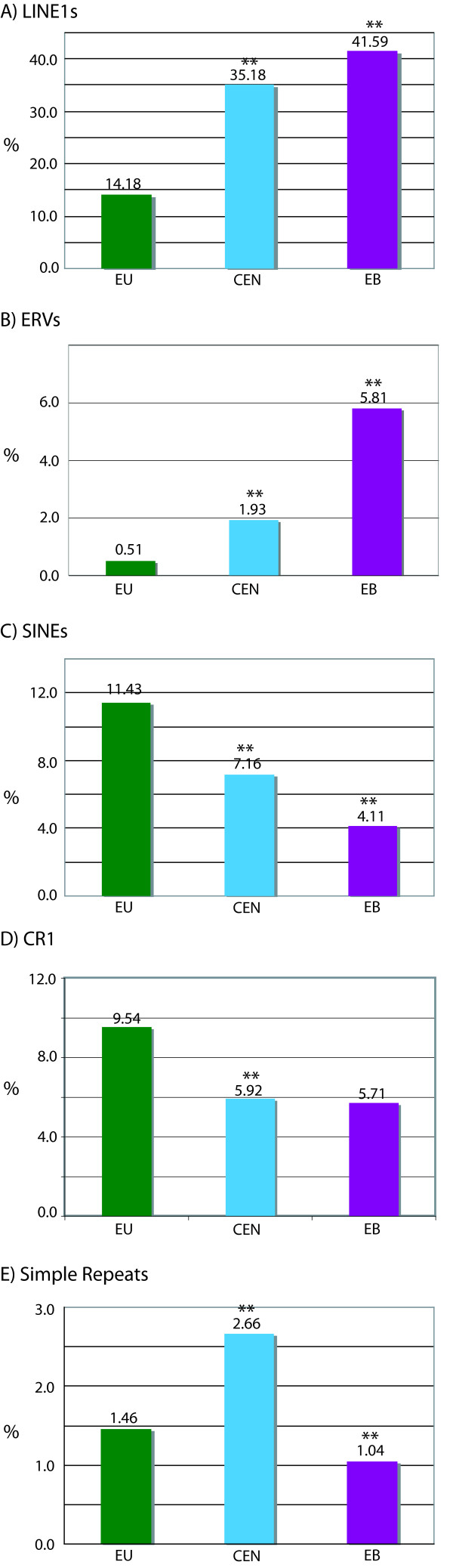
**Quantification of interspersed repeats**. Percentage of sequence predicted to be (A) LINE1s, (B) endogenous retroviruses (ERVs), (C) SINE elements, (D) CR1s, and (E) Simple Repeats in the tammar by region – EU, CEN, and EB. (** statistically significant difference from EU)

Both the CEN and EB regions have many repeat types in common, most notably a significant enrichment of both endogenous retroviruses (ERVs) and LINE1s (L1s) while being relatively devoid of SINEs (Figure 4A–C). This is also visually apparent in the annotation of these sequences (Figure 3). The total L1 average for each region is 41.59% at the EB, 35.18% in the CEN and only 14.18% in the EU region (Figure 4A). Some classes of L1s are nearly exclusively found in the EB (L1-3_MD, L1-2_MD, and L1_RN) but represent the minority of this general class. Two of the most abundant L1s (L1-3_ME, L1-3A_ME) are found in all three regions but differ in copy number considerably with the majority found in the EB, averaging roughly 19.5 elements per 100 kb (see Additional File 2). This is significantly more than the 13.4 elements per 100 kb found in the CEN regions and significantly more than the 4.8 per 100 kb in the EU (*p *< 0.001).

**Figure 5:**
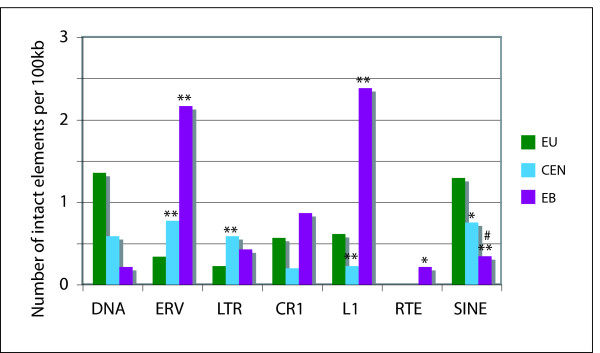
**Number of intact repeats (95% or more of consensus sequence length) estimated for every 100 kb by region (EU, CEN, EB)**. (** statistically significant difference from EU, # statistically significant difference from EU and CEN)

Of the 71 L1 elements (of all types) with 90% or greater identity to their consensus sequences, 63% (45) are found in the 972 kb derived solely from the EB and CEN regions (approximately one-third of the 2.7 Mb of sequence analyzed). To further refine this apparent region bias, repeats 95% or more of their consensus length were identified as intact and further quantified. The number of repeats was normalized for discrepancies in region size among EB, CEN, and EU by estimating the number of intact elements for every 100 kb (Figure 5; see Material and Methods). Intact L1s were primarily found within the EB region with almost 2.4 elements for every 100 kb. There were far fewer in the CEN (.23) and EU (.62) regions. These observations suggest that the EB is enriched for intact L1s that have likely been very recently active and may contribute to the instability of this region.

Content variation between these three genomic domains was not restricted to LINEs. ERVs make up 5.81% of the EB region and 1.93% of the CEN compared to 0.51% in the EU region (Figure 4B). The EB contains a wider variety of viral elements than the CEN regions. The most conserved and abundant ERV in both regions is the virus MERVK1-I as identified by Censor. This element is of particular interest because it is in fact a mis-annotated KERV sequence, the virus sequence used in the initial BAC screens and previously identified [6]. MERVK1-I is only a portion of the complete KERV-1 sequence (Ferreri, O'Neill personal communication). Repbase also identifies solo KERV-LTR sequences (ERVII_ME_LTR and MERVK1_LTR). There are an average of 1.89 KERV and 1.07 KERV solo LTR sequences found in each 100 kb of the EB. The CEN regions contain 0.78 copies (per 100 kb) of both KERV and KERV LTRs while the EU regions contain only 0.23 copies (per 100 kb) of KERV LTRs and no non-LTR KERV sequence (see Additional File 3). There is a general enrichment of many different ERVs in both the EB and CEN regions (Figure 4B) with KERV being the most abundant and conserved and the only ERV present in its full length.

The euchromatic (EU) regions, while being relatively devoid of ERVs and L1s contain an average number of repeats (41.14%), on *par *with estimates of human repeat content of 40.3% and slightly higher than previous estimates for the tammar wallaby of 37.0% [23]. The most common repeat class found in the EU regions are the short interspersed nuclear elements (SINEs), comprising 11.43%, and the non-LTR retrotransposon CR1s that make up approximately 9.54%. This varies dramatically from the EB at 4.11% SINEs and 5.71% CR1s and the CEN regions at 7.16% and 5.92% respectively (Figure 4C–D). The SINE content of the CEN regions, while not as dramatically different from EB and EU regions as for ERVs, carries a SINE content midway between EU and EB; however, the CEN SINE content is still significantly different from either EU or EB (p = 0.0026 and p = 0.012). Full-length copies of SINEs are also more frequently found in the EU (Figure 5). The CR1 content when averaged appears very different in the EB compared to the EU but only has a *p*-value = 0.212 due to the disparate distribution of this class on the three BACs analyzed. DNA transposons are more prevalent in the EU (1.33%) than the EB (0.45%) and CEN (0.59%). One exception to this is the DNA transposon CHARLIE1B from the hAT class of repeats which is almost exclusively limited to the CEN regions (data not shown).

Ruiz-Herrera et al (2006) have shown the reuse of EB between species as well as a correlation between fragile sites and tandem repeats within these locations [25]. We have found more simple repeats (including tandem repeats) at the CEN regions than either EU or EB and, interestingly, the fewest at the EB (Figure 4E).

This analysis shows a general enrichment of ERVs and L1s at both a conserved EB and centromeric regions of the tammar wallaby genome. More specifically, we have shown that the L1 elements, L1-3_ME and L1-3A_ME, as well as the endogenous retrovirus KERV and its solo-LTRs are both more abundant and more intact at the EB and CEN compared to the genic regions (EU) examined.

### Conserved evolutionary breakpoint between tammar wallaby 1q and human 14q32.33

To examine these regions further, the sequences of the 8 BACs (Figure 1 and 3) were examined for possible protein coding regions. The sequences were masked of repeats and analyzed with the two gene prediction programs, GenScan and Genemark.hmm-E. Predicted coding regions were analyzed using the BlastN, BlastX (NCBI) and BLAT (UCSC) analysis programs (see Methods). Coding regions were predicted in each of the three chromosomal domains (CEN, EB and EU) at approximately equal frequencies. None of the predictions in the CEN region were identifiable as known coding regions. Conversely, both the EB and EU contain predicted coding regions with some sequence identity, at least at the protein level, for known genes (see Additional File 4).

Interestingly, the EU BAC A8 carries a predicted gene with high nucleotide homology (83.8% identity) to the human gene Transmembrane protein 179 (TMEM179) (Gene Accession # Q6ZVK1). TMEM179 is located on human 14q32.33 approximately 1 Mb upstream from the immunoglobulin heavy chain (IGH) region, which has been involved in translocations in multiple myelomas and plasma cell leukemias [26]. Human 14q32.33 has also been identified as an EB [25]. The localization of A8 adjacent to the 1q EB clones and the activity of IGH in human cancer compelled a closer examination of the IGH orthologous locus in human with respect to conserved features.

**Figure 6:**
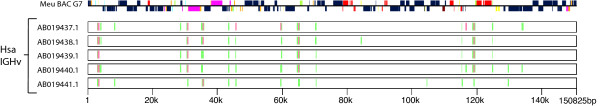
**Multipipmaker alignment of tammar BAC G7 (1q EB) aligned to the 5 clones that make up the human IGHv contig (bottom)**. Alignments performed with repeats masked. Above, map of G7 from Figure 3 showing repeat distribution relative to regions of 14q32.33 orthology.

The immunoglobulin heavy chain locus is approximately 1.25 Mb on human chromosome 14 and consists of both a constant and variable region [27]. The IGHv region is the most distal 1 Mb of human 14q, the assembled BAC contig of which consists of 5 clones. Sequence alignments using MultiPipMaker with repeats masked were performed comparing each tammar EB clone (I6, G7 and B9) with clone sequences used in the assembly of human chromosome 14q32.33 [28], spanning IGHv to TMEM179. While there was no identity between these segments and I6, or B9, several regions of the EB clone G7 had significant alignment across the terminal segments of 14q32.33, representing only the IGHv region (Figure 6). Each of these G7 sequences showed significant identity to the IGHv region of many species including human, mouse, chimp and opossum (see Additional File 5). The alignments with tammar G7 were of sufficient length and nucleotide identity to identify it as orthologous to the IGHv region. These alignments at first glance appear disrupted but when examined in the context of the repeats in this region as determined by our annotations (Figures 3), it is clear that the orthologous regions fall between the repeats (Figure 6). In addition, the alignments of G7 to five different, contiguous clones from human 14q32.33 (Figure 6) are indicative of the segmental duplications this region of human chromosome 14 has experienced [28]. It is not known at this time, however, whether segmental duplications are a shared feature between this region of the human genome and the tammar EB region given the lack of full, contiguous sequence for this region of the tammar genome.

**Figure 7:**
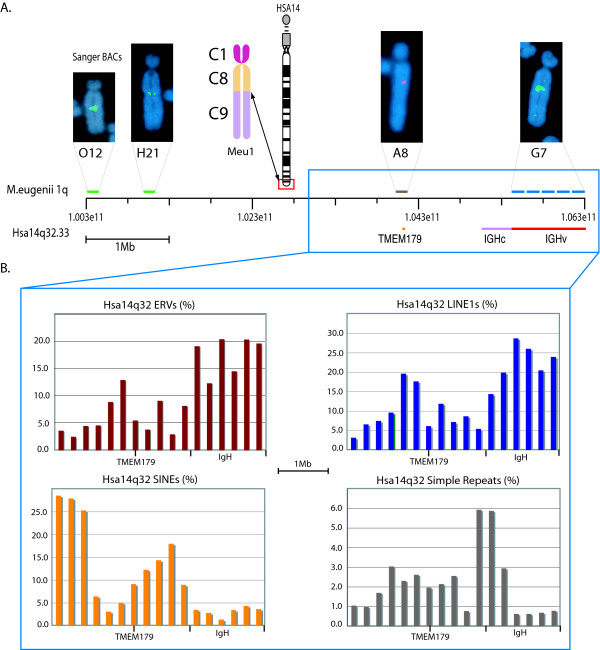
**Map of Hsa14q32 and Meu 1q**. (A) Map of *M. eugenii *1q compared to the orthologous human 14q32.33 showing tammar BACs FISH mapped to the EB region of 1q and their relative position on 14q. BACs O12 and H21 have been identified by the Sanger Institute to be orthologous to Hsa14q32. BAC A8 and G7 were identified by screening the *M. eugenii *BAC library with KERV. BAC A8 contains a predicted protein with high homology to human TMEM179. BAC G7 contains regions with high identity to the entire region of the human immunoglobulin heavy chain variable region (IGHv). (B) The density of ERVs, LINE1s, SINEs, and simple repeats in the most distal 3.4 Mb of 14q32 in increments of 200 kb, including the IGH region and TMEM179.

Assemblies of BACs for the tammar wallaby across a region orthologous to approximately 3 Mb upstream from TMEM179 on human 14q are publicly available (Sanger Institute). Two clones (H21 and O12) from this contig were localized to tammar wallaby metaphase chromosomes using fluorescence *in situ *hybridization to verify orthology. Both clones hybridized to tammar 1q and verify this region of 1q as orthologous to human 14q (Figure 7A). While G7 and A8 align to the human contig for 14q32.33 (Figure 7A, right), the other two tammar EB clones (I6 and B9, Figure 1B) had no significant identity with the IGH locus. It is likely these BACs lie distal to the break and are not represented in human 14q, however the resolution of metaphase FISH did not allow for a finer map location with respect to the 14q orthologous region.

### Enrichment of LINEs and ERVs at human 14q32

Given the orthology between the 1q EB in the tammar wallaby and human 14q32, we tested whether the observed enrichment of specific repeats was conserved in divergent mammalian lineages. Our data shows that this breakpoint represents an EB that has been conserved at least since the last shared common ancestor of the Eutherian and Metatherian lineages, approximately 147MYA [16]. Moreover, this is an EB that is still unstable in humans in that it is a fragile site that undergoes rearrangement during disease progression [26] and has been observed to form a neocentromere [29]. Across human 14q32.33 are regions orthologous to both EB and EU regions within tammar.

The pattern of repeats in human14q32.33 mirrors that observed between the EU and EB regions of tammar wallaby (Figure 7B). From TMEM179 to just before the IGH constant region (IGHc), the region orthologous to the tammar EU represented by BAC A8, there is an abundance of SINE elements and relative paucity of both LINE1s and ERVs. The human sequence beginning at IGHc and continuing to the end of this region (IGHv) shows a dramatic increase in LINE1 and ERV content and a drastic decrease in SINEs, analogous to that observed for the tammar EB.

**Figure 8:**
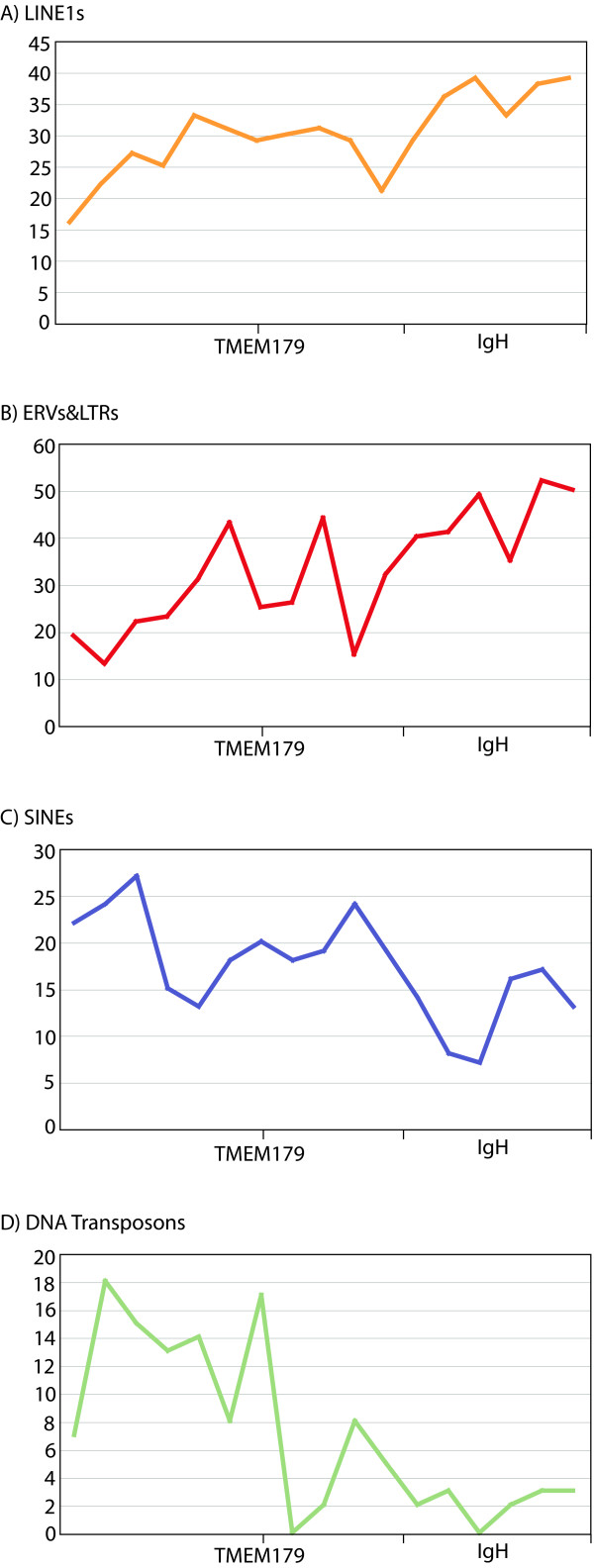
**The diversity of (A) LINEs, (B) ERV/LTRs, (C) SINEs and (D) DNA transposons in Hsa 14q32.33**. Shown is the number of different types of elements from each class identified by Censor spanning Hsa14q32.33 in increments of 200 kb.

We have examined the repetitive elements across the cytological band 14q32.33 to determine if this distribution represents an expansion of a small group of repeats or an enrichment or absence of the particular repeat classes in general. We found that where there was an abundance of an element class it reflected an increase in diversity as well as number (Figure 8). For instance, the number of ERVs and LINE1s dramatically increases, as does the diversity of those element types, with approximately 50 different ERVs and almost 40 different LINE1s identified in the most distal portion of 14q32.33 (IGHv) and less than 20 of each type identified in the more proximal region (EU) (Figure 8A and 8B). The reverse is true for SINEs with as many as 27 varieties in the EU region compared to as few as 7 in the EB (Figure 8C). DNA transposons, despite the fact that their quantity did not vary significantly, carry a diversity across the region resembling that of SINEs. This observed diversity is even more pronounced with up to 18 types in the EU compared to 3 or less in the EB (Figure 8D). CR1 diversity (data not shown) had slightly more variety in the EU than the EB, though this may or may not be significant as there are only 3 different CR1s throughout the region as classified by Censor. Simple repeats were not included in this portion of the analysis as they are too varied to easily group and analyze.

**Figure 9:**
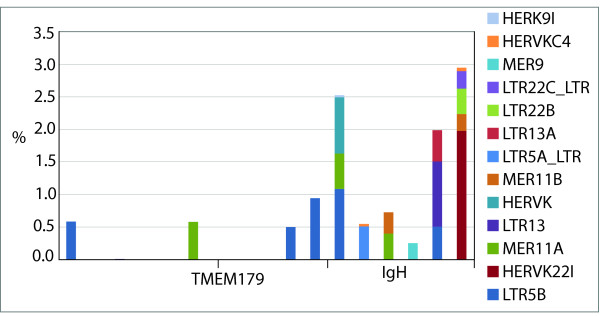
**The percent of ERV2 type repeats identified by Censor spanning Hsa14q32.33 in increments of 200 kb showing the relative contribution of each specific ERV identified**.

Interestingly, the ERV2 class of endogenous retroviruses (as identified by Censor) was found almost exclusively at the IGH region of 14q32.33 (Figure 9). All of the members found belong to the HERVK class of ERVs. HERVKs are phylogenetically related to mouse mammary tumor viruses (MMTVs) in mice [30]; KERV has been shown to be most closely related to this group [6,18].

Human 14q32.33 is found at the telomere end of human chromosome 14. To determine if this repeat pattern is a feature of telomeric regions rather than that of EBs, two human telomeres not previously identified as EBs [25] were chosen at random (11q and 16q) and a repeat analysis was performed on the most distal 3 Mb of each. The dramatic enrichment for LINE1 and ERVs was not observed for either telomere (see Additional File 6). A similar analysis was performed on the IGH region of the South American opossum (*Monodelphis domesticus*) (see Additional File 7). Though a similar trend is seen we were unable to determine statistical significance due to many large gaps in the *M. domesticus *assembly. However, there is a very dramatic enrichment of L1s and ERVs across the entire 4 Mb examined including the IGH region.

## Discussion

The recent availability of large amounts of genome sequence from diverse taxa has allowed for high-resolution mapping of syntenic chromosomal segment order in efforts to understand the evolutionary trajectory of specific genomic regions. Murphy et al [12] examined orthologous genomic sequences of syntenic blocks among a broad array of eutherian species and found that breakpoint locations are often reused between divergent species and that these sites strongly correlated with centromere locations in several species. Ruiz-Herrera et al [25] examined the Murphy et al. dataset and found that not only is there a link between breakpoints and centromeres in karyotype evolution, but EBs also coincide with fragile sites and chromosomal breakpoints identified in human cancers [25,31]. These studies suggested that EBs might continue to carry "signals" of both past breakpoint activity as well as a propensity for further instability under cellular stress; however, these studies did not examine EB sequences in a phylogenetic context.

More recently, mapping the trajectory of chromosome segments along species phylogenies in marsupial lineages has shown that breakpoint reuse often coincides with centromere emergence [3,17], lending support to the hypothesis that EB serve as latent centromeres [22]. Thus, we can predict that the EBs characterized as latent centromeres might retain common sequence features between divergent taxa. Marsupialia offers an ideal system to study genomic rearrangements and breakpoint reuse; this infraclass represents one of the most well characterized mammalian lineages with respect to chromosome arrangement. Over 70% of extant species have been karyotyped ([32,33] and reviewed in [7]) and the chromosome trajectories of many families, genera and species have been determined (e.g. [3,17]). With comparatively little marsupial sequence data available, cross-species reciprocal chromosome painting has been effectively used to delineate conserved chromosome segments (orthologous chromosome blocks) and to identify convergent breakpoint reuse [3,17].

Our study utilizes a comparative sequencing approach to test the hypothesis that EBs and CEN share specific sequence features and that such features are retained during periods of genomic instability and species evolution. We have identified specific interspersed repeats, endogenous retroviruses (ERVs) and long interspersed nuclear elements (L1s), enriched in EBs and CEN. These particular groups of repetitive elements (ERVs and L1s) are also found at several breaks of synteny between human and gibbon [34] as well as two breakpoints examined between human and chimp [35]. We also show that the interspersed repeat distribution of CENs and EBs differs dramatically compared to that of a previously analyzed euchromatic region (the *CFTR *locus) [23]. In human tumor cell lines, chromosome 3 shows regions of recurrent instability. The distribution of repeats at these loci has a very similar increase of both L1 and ERV elements [36].

Through BAC mapping and comparative sequence analyses, we show that the EB on tammar 1q is orthologous to human 14q32.33. This locus has been identified as an EB [25], is known to undergo translocations associated with cancer [26], and has been identified as a neocentromere [29]. We have analyzed repeat content of the tammar EB and surrounding EU and compared them to the repeat distribution of the orthologous human region, 14q32.33, including the immunoglobulin heavy chain region (IGH). As in tammar, the human orthologous EB carries a significant enrichment of ERVs and L1s, with frequencies of both sequences similar to that observed for tammar CEN. These data suggest that repeat content defines distinct chromosome domains and is a conserved feature of mammalian genomes. Moreover, CEN and EBs are enriched for both ancient ERV and recent L1 activity, indicating these regional domains, and subsequent instability that manifests as chromosome rearrangement or centric shifts, is directly linked to the activity of mobile DNA. It is worth noting that the primary satellite sequence found in the *Cetacea *is derived from an ancestral mammalian L1 element [37].

The enrichment of ERVs, and specifically HERV-K retrotransposons, in 14q32.33 is of particular interest given that this class contains primate specific lineages of elements and thus must be recently derived. HERV-K retroviruses consist of 10 different families of human MMTV-like elements, denoted as HMLs 1–10 [38]. Some of these families, such as HML-2, are characterized by recent activity in the genome and contain intact open reading frames (ORFs) that encode functional proteins [39,40], while other families, such as HML-3 and HML-5, have not been active for tens of millions of years [30,38]. The prominent element in the human breakpoint examined is denoted in Repbase as HERV-K22, an HML-5 element [38]. Last active prior to the split of Old World and New World primates, this element would have integrated into this location long before hominoid divergence, and thus has been retained despite breakpoint activity in this region. Moreover, the integration of an HML-5 member in this region parallels an integration of another ancient HERV-K related element, KERV, in the orthologous region within the Metatherian lineage (Meu1q).

KERV, while ancient in origin, has retained a cellular function in active centromeres through recruitment of specific centromere proteins and production of novel small RNAs in marsupial and eutherian lineages [20]. Likewise, transcription of HERV-K [41] elements has also been retained, although functional coding sequences for either class of elements have not been identified nor has any involvement with cellular function been examined. Thus, not only is there a tight correlation between EBs and CEN as regional domains involved in genome rearrangement, instability and karyotypic evolution, there is a tight correlation between specific sequences found in these regions (i.e. HERV-K type elements). Two scenarios may explain the presence of these elements at orthologous EB: either HERV-K replaced KERV elements within a eutherian ancestor at the region orthologous to 14q32.33, or the KERV and HERV-K elements independently integrated into orthologous EB. Understanding the integration preference sites for each respective class may shed light on the order of integration events.

Given the predisposition of the EB on Meu1q and Hsa14q32.33 for continuous rearrangement through double-strand breaks and ENC formation within both marsupials and humans, the coincidence of specific classes of retroelements at these regions implies they may be integral to the underlying mechanism for prolonged instability. A recent study of double-strand repair mechanisms in yeast showed that those breaks that give rise to chromosome aberrations were repaired by homologous recombination (HR) between nonallelic Ty retrotransposons [42]. In light of the finding that HR between nonallelic repeat elements contributed to a large portion of the structural variation in the human genome [43,44], it is intriguing to consider that sustained activity of retroelements, not necessarily through transposition, but rather through an inherent propensity for HR between elements at distant genomic locations may contribute to both the evolutionary novelty of the genome but also to its innate instability.

## Conclusion

With many genome assemblies available, it is clear that breakpoints in the genome are nonrandomly distributed, frequently reused in karyotypic evolution and often involved in diverse disease states. Phylogenetic analyses of whole genome sequence has shown that breakpoints are frequently the location of ancient centromeres and novel centromere formation [12]. Our study examines the relationship between evolutionary breakpoints and centromeres in the tammar wallaby, *Macropus eugenii*. We have examined sequence from a breakpoint (Meu1q) that has been reused throughout the karyotypic diversification of the Marsupialia. These data were compared to sequence from three centromeres (Meu2, Meu5, and Meu7) and a euchromatic coding region. We hypothesized that breakpoints would resemble centromeres in genomic content given the prevalence for centromeres to occur at conserved breakpoints in marsupials and found this to be the case; both regions carried a statistically significant enrichment of certain ERV and L1 elements. Furthermore, we extended our study and examined the human ortholog of the tammar breakpoint, Hsa14q32.33. This locus has been known to be involved in translocations in many diseases, including multiple myelomas and plasma cell leukemias. Moreover, a neocentromere has also been described at this region. Our analysis of the human ortholog shows a drastic increase in the number of L1s and ERVs and a depletion of SINE elements, sequence features conserved at the tammar breakpoint and centromeres. Our results show these sequence classes have been retained at this region since the divergence of marsupial and eutherian mammals despite replenishment with lineage-specific elements. Thus, continued activity of these classes of elements may contribute to the instability observed at these locations and may serve as an indicator of centromere potential.

## Methods

### FISH

Fluorescence *in situ *hybridization of BAC DNA was performed as per [18]. Location to breakpoints between conserved chromosome segments was confirmed by chromosome painting as per [18].

### Sequence analysis

Tammar BACs B9, G7, I6, G17, B18, M7, A8, and J6 were sequenced at the NIH Intramural Sequencing Center (NISC), as described previously [45]. Additional tammar BAC sequence (from the genomic region containing the *CFTR *gene) was obtained from NCBI  (see Additional Table 1 for complete list of accession numbers of BACs used. Human 14q32 sequence was obtained through the UCSC genome browser .

Gene predictions were made with Genscan  with default settings (suboptimal exon cutoff = 1) and source sequence set as human for all analyses. Additional gene predictions were made using GeneMarkHMM . The GeneMark-E with GeneMarkHMM-E for eukayotic genomes was used with human chosen as the reference species. All predictions were performed on sequences masked for repeats by Censor (see below). Lengths of coding regions from each program were then averaged and used to determine the percent of each region. All predicted exons were analyzed using NCBI's BlastN and BlastX  and UCSC's BLAT .

All repeat analyses were performed using both Censor [24] and Repeatmasker . Both programs resulted in nearly identical data sets. Sequence source for Censor was *Monodelphis domestica *(the South American opossum) when analyzing marsupial sequence and *Homo sapiens *for human sequences. Repeatmasker default settings (search engine = cross-match) were used with sequence source set to either mammalia or human for marsupial or human sequences respectively. The number of each class and individual type within each class of repeat was quantified as both number of occurrences and as a percentage of sequence for each clone. Percentages were determined by totaling the number of nucleotides for each repeat class divided by the region length. To determine the number of each repeat class for every 100 kb, the number of each repeat as reported by Censor was determined for each region and then divided by the total length of that region and multiplied by 100 k. Intact repeats were determined as follows; all repeats 95% or more of their consensus length were identified. These were then divided by clone length and multiplied by 100 k. All repeats and gene predictions were fully annotated using the annotation program Artemis 

Multipipmaker  and Codon Code Aligner  was used to align larger clones to identify possible contigs among the selected tammar BAC sequences and determine possible orthology between tammar and human. Regions of identity were then confirmed using NCBI's blast algorithms. All alignments were performed with interspersed repeats masked by Censor.

## Competing interests

The authors declare that they have no competing interests.

## Authors' contributions

ML wrote the manuscript and performed the analyses, DC performed several of the FISH experiments, NISC Comparative Sequencing Program and EG performed the BAC sequencing and assembly, MO and RO conceived the study and wrote the manuscript.

## Supplementary Material

Additional file 1 **BAC clones analyzed with accession numbers**. All clones have been analyzed for interspersed repeat content. Tammar clones indicated with (*) have been mapped to metaphase chromosomes with fluorescence in situ hybridization (FISH). Click here for file

Additional file 2 **List of interspersed repeats whose average copy number varied significantly**. List of interspersed repeats whose average copy number varied significantly (*p=*0.01) between EU, CEN and EB regions in the tammar genome. Within columns, bars in cells depict relative number of copies visually. Red bars are those enriched in EB only, orange are those enriched in EB and CEN but not EU regions. Blue bars in cells highlight those repeats found more in EU and not in CEN nor EB. Green bars show elements found more abundantly in CEN regions. *p*-values from a standard t-test indicating the level of significance (green cells *p=* 0.01, red cells *p=* 0.05). Click here for file

Additional file 3 **Average number of KERV-1 elements**. Average number of KERV-1 elements, both internal coding and LTR, estimated for every 100kb. Click here for file

Additional file 4 **List of predicted genes in tammar BACs**. List of predicted genes in tammar BACs showing percent identity and their respective NCBI accession numbers and origin species (MD= *Monodelphis domestica*; HSA= human). Identities shown in black represent translated identities to known proteins by BLASTX alignment. Transmembrane protein 179 (TMEM179) has high nucleotide identity using UCSC’s BLAT alignment algorithm. Click here for file

Additional file 5 ***M.eugenii* BAC G7 with identity to immunoglobulin heavy chain variable region**. Regions of *M.eugenii* EB BAC G7 with identity to immunoglobulin heavy chain variable region (IGHv) in various species as identified with NCBI’s BLASTN. Click here for file

Additional file 6 **Repeat distribution for 2 telomeres (11q and 16q) that are not known to be evolutionary breaks**. Shown are the percent of sequence identified as ERV (A, D), L1 (B, E), or SINE (C, F) for the terminal 4Mb of each chromosome. Click here for file

Additional file 7 ***Monodelphis domesticus* repeat distribution of the IGH region**. *Monodelphis domesticus *repeat distribution of the IGH region and approximately 1Mb of either side. Shown are the percent of sequence identified as (A) ERV, (B) L1, (C) SINE, (D) Total repeats, and (E) Simple repeats. This region has several gaps in the genome assembly. (F) Shown are the percent of ambiguous bases found in the region. Click here for file
