# Supplementary material for: Distinct retroelement classes define evolutionary breakpoints demarcating sites of evolutionary novelty
Source: BMC Genomics. 2009 Jul 24;10:334. doi: 10.1186/1471-2164-10-334 (PMC2736999; doi:10.1186/1471-2164-10-334)
Supplement: Additional file 1 — BAC clones analyzed with accession numbers. All clones have been analyzed for interspersed repeat content. Tammar clones indicated with (*) have been mapped to metaphase chromosomes with fluorescence in situ hybridization (FISH). [file 1471-2164-10-334-S1.pdf]

M.Eugenii	Clone Name	Accession #
**Break of Synteny**		
B9 *	ME_Kba-581B9	AC166214
G7 *	ME_Kba-599G7	AC169004
I6 *	ME_Kba-32I6	AC166213
**Pericentric**		
B18 *	ME_Kba-587B18	AC167934
G17 *	ME_Kba-586G17	AC174420
M7 *	ME_Kba-21M7	AC166212
**Euchromatic**		
A8 *	ME_Kba-583A8	AC166216
J6 *	ME_Kba-583J6	AC166217
**CFTR**		
	ME_KBa-289E24	AC145042
	ME_KBa-528O13	AC145409.3
	ME_KBa-169G7	AC145407.3
	ME_KBa-363H17	AC145841.3
	ME_KBa-232I1	AC145250.3
	ME_KBa-60J17	AC145183.3
	ME_KBa-210L14	AC145408.3
	ME_KBa-103M1	AC145249.3
	ME_KBa-71M6	AC145184
	ME_KBa-210M21	AC145041.3
**Sanger Hsa14q32 orthologs**		
H21 *	MEKBa-61H21	CU019600
O12 *	MEKBa-325O12	CR933563
H.Sapiens		
**IGHv region**		
	-	AB019437.1
	-	AB019438.1
	-	AB019439.1
	-	AB019440.1
	-	AB019441.1
