# Supplementary material for: Distinct retroelement classes define evolutionary breakpoints demarcating sites of evolutionary novelty
Source: BMC Genomics. 2009 Jul 24;10:334. doi: 10.1186/1471-2164-10-334 (PMC2736999; doi:10.1186/1471-2164-10-334)
Supplement: Additional file 2 — List of interspersed repeats whose average copy number varied significantly. List of interspersed repeats whose average copy number varied significantly (p=0.01) between EU, CEN and EB regions in the tammar genome. Within columns, bars in cells depict relative number of copies visually. Red bars are those enriched in EB only, orange are those enriched in EB and CEN but not EU regions. Blue bars in cells highlight those repeats found more in EU and not in CEN nor EB. Green bars show elements found more abundantly in CEN regions. p-values from a standard t-test indicating the level of significance (green cells p= 0.01, red cells p= 0.05). [file 1471-2164-10-334-S2.pdf]

Number of elements per 100kb

Repeat Name (Repbase name)	Region			T-test p-value		
	EU	CEN	EB	EU v. CEN	EU v. EB	EB v CEN
DNA						
DNANA5_MD	1.58	0.59	0.22	0.100830	0.029859	0.1869505
DNA/hAT						
CHARLIE1B	0.11	0.39	0.00	0.045264	0.241805	0.0580583
hATA_ME	0.06	0.00	0.65	0.317469	0.036741	0.1869505
ERV						
ERV7b_MD_I	0.00	0.00	0.22		0.020046	0.1869505
ERVB1_1-I_RN	0.00	0.00	0.22		0.020046	0.1869505
ERVB2_2-I_RN	0.00	0.00	0.22		0.020046	0.1869505
PRIMA4_I	0.00	0.00	0.22		0.020046	0.1869505
ERV/ERV1						
ERV8_MD_I	0.00	0.00	0.43		0.020046	0.1869505
ERV20_MD_I	0.00	0.00	0.22		0.020046	0.1869505
ERV48_MD	0.00	0.00	0.43		0.000267	0.0580583
MER50I	0.00	0.00	0.22		0.020046	0.1869505
MER83AI	0.00	0.20	0.22	0.020046	0.020046	0.5
PABL_BI	0.00	0.00	0.22		0.020046	0.1869505
RLTR4I_MM	0.00	0.00	0.22		0.020046	0.1869505
ERV/ERV2						
ERV23_MD_I	0.00	0.00	0.22		0.020046	0.1869505
ERVII_ME_LTR	0.00	0.00	1.09		0.000036	0.033383272
IAPEY3_I	0.00	0.00	0.22		0.020046	0.1869505
IAPLTR1a_I_M	0.11	0.39	0.43	0.045264	0.101340	0.5
MERVK1_I	0.00	0.78	1.95	0.000267	0.000000	0.0658888
MERVK1_LTR	0.23	0.78	1.09	0.029859	0.008266	0.3707605
ERV/ERV3						
ERVL-E	0.00	0.20	0.00	0.020046		0.1869505
MLT2A1	0.00	0.20	0.00	0.020046		0.1869505
Interspersed Repeat						
UCON1	0.00	0.20	0.00	0.020046		0.1869505
Non-LTR						
WRETRO	0.00	0.39	0.43	0.020046	0.000267	0.5
Non-LTR/CR1						
L2A	3.90	1.95	0.87	0.115045	0.020727	0.0505958
Non-LTR/CR1						
L2B_ME	17.99	7.81	7.60	0.003234	0.002922	0.3954223
Non-LTR/L1						
L1_RN	0.00	0.00	0.22		0.020046	0.1869505
L1-1_DV	0.00	0.39	0.43	0.000267	0.020046	0.5
L1-1_ME	2.09	3.90	5.65	0.035474	0.006804	0.2219694
L1-2_MD	0.11	0.20	0.43	0.276365	0.045264	0.2592593
L1-2_ME	1.47	7.61	7.17	0.000002	0.000641	0.3337461
L1-3_MD	0.51	0.39	2.82	0.455812	0.000742	0.034857904

L1-3_ME	0.85	3.90	4.99	0.000075	0.000015	0.2939572
L1-3A_ME	3.96	9.56	14.55	0.000473	0.000107	0.153173
L1-4_MD	3.00	4.29	4.78	0.041796	0.068434	0.5
L1-4_ME	1.81	3.71	6.51	0.029149	0.006837	0.2865063
L1-5_MD	0.28	0.98	0.00	0.020992	0.156508	0.0658888
L1C-OC	0.00	0.20	0.00	0.020046		0.1869505
L1HS	0.06	0.00	0.22	0.317469	0.143604	0.1869505
L1N1_MD	2.55	3.51	7.38	0.195305	0.012628	0.1273347
L1-Y_CF	0.00	0.00	0.22		0.020046	0.1869505
**Non-LTR/RTE**						
BDDF2	0.00	0.00	0.22		0.020046	0.1869505
RTE0_MD	7.35	2.73	2.39	0.025023	0.014779	0.3748234
RTE0_ME	3.28	0.98	0.22	0.051966	0.012450	0.1150998
RTE-1_MD	0.06	0.00	0.87	0.317469	0.005583	0.1025532
RTE-2_ME	0.57	1.76	1.74	0.013606	0.016295	0.4360791
**Non-LTR/SINE**						
B2	0.00	0.20	0.00	0.020046		0.1869505
MAR1_MD	8.31	6.05	2.39	0.228441	0.002002	0.00416756
MAR1b_MD	2.15	1.37	0.43	0.238304	0.020992	0.044504671
MAR4_MD	0.00	0.00	0.43		0.020046	0.1869505
MIR3	6.22	3.51	2.39	0.084614	0.029984	0.2862263
THER1	12.16	6.24	1.74	0.004443	0.000011	0.005594415
THER1_MD	4.69	2.93	1.95	0.172070	0.029079	0.0791512
THER2_MD	7.64	5.27	1.30	0.273311	0.004807	0.1063331
WALLSI2	0.00	0.00	0.22		0.020046	0.1869505
WALLSI3	1.47	0.20	0.87	0.047474	0.220613	0.1254077
WALLSI4	7.52	4.29	1.74	0.082935	0.002638	0.0787418
MIRb	4.19	3.12	1.74	0.345055	0.060192	0.0958722
THER2_ME	10.86	7.42	1.52	0.264998	0.004611	0.0921016
WALLSI2	1.64	0.98	0.22	0.180538	0.005659	0.1150998
