# Supplementary material for: Distinct retroelement classes define evolutionary breakpoints demarcating sites of evolutionary novelty
Source: BMC Genomics. 2009 Jul 24;10:334. doi: 10.1186/1471-2164-10-334 (PMC2736999; doi:10.1186/1471-2164-10-334)
Supplement: Additional file 4 — List of predicted genes in tammar BACs. List of predicted genes in tammar BACs showing percent identity and their respective NCBI accession numbers and origin species (MD= Monodelphis domestica; HSA= human). Identities shown in black represent translated identities to known proteins by BLASTX alignment. Transmembrane protein 179 (TMEM179) has high nucleotide identity using UCSC’s BLAT alignment algorithm. [file 1471-2164-10-334-S4.pdf]

Meu. BAC	Predicted Gene – BlastX results	% Identity	Gene Accession
A8	Transmembrane protein 179	**83.8 (HSA)**	Q6ZVK1
B9	Ribosomal protein L13	85 (HSA)	AAH66320
G7	eukaryotic translation elongation factor 1 alpha 1 variant	91 (MD)	BAD96750
I6	Phosphofurin acidic cluster sorting protein 2 (PACS-2) (PACS1-like protein).	82 (MD)	Q86VP3
J6	LIM domain binding 2, isoform CRA_b	62 (MD)	EAW92766

BLAT (UCSC-nucleotide identity) **BlastX** (NCBI-protein similarity)
