# Supplementary material for: Distinct retroelement classes define evolutionary breakpoints demarcating sites of evolutionary novelty
Source: BMC Genomics. 2009 Jul 24;10:334. doi: 10.1186/1471-2164-10-334 (PMC2736999; doi:10.1186/1471-2164-10-334)
Supplement: Additional file 5 — M.eugenii BAC G7 with identity to immunoglobulin heavy chain variable region. Regions of M.eugenii EB BAC G7 with identity to immunoglobulin heavy chain variable region (IGHv) in various species as identified with NCBI’s BLASTN. [file 1471-2164-10-334-S5.pdf]

G7 Range	Species	% nucleotide identity (IGHv)	Length of alignment
2898-3694	H.sapiens	82	179
2898-3695	L.pacos	82	179
2898-3693	M.domesticus	84	304
2898-3697	O.anatinus	88	241
2898-3696	T.vulpecula	84	150
30557-30973	H.sapiens	93	43
30557-30972	M.domesticus	83	95
30557-30976	M.mulatta	89	46
30557-30974	M.musculus	91	46
30557-30978	P.troglodytes	89	46
30557-30977	R.norvegicus	89	46
30557-30975	T.vulpecula	84	66
34945-35330	H.sapiens	89	58
34945-35332	M.mulatta	89	49
34945-35333	P.troglodytes	86	58
34945-35331	R.norvegicus	90	50
64663-65420	C.familiaris	82	131
64663-65422	H.sapiens	90	60
64663-65419	I.macrourus	83	117
64663-65417	M.domesticus	80	251
64663-65423	M.musculus	84	95
64663-65421	O.anatinus	82	130
64663-65418	T.aculeatus	83	118
64663-65416	T.vulpecula	86	294
118737-119498	C.auratus	82	160
118737-119495	C.dromedarius	86	107
118737-119501	C.migratorius	89	77
118737-119492	H.sapiens	83	171
118737-119502	I.macrourus	82	144
118737-119496	L.granatensis	91	78
118737-119499	L.pacos	94	59
118737-119490	M.domesticus	81	244
118737-119494	M.mulatta	83	171
118737-119491	O.cuniculus	91	80
118737-119493	P.troglodytes	83	171
118737-119497	R.norvegicus	95	60
118737-119500	T.aculeatus	82	159
118737-119489	T.vulpecula	87	308
